# Effect of Pneumococcal Vaccine on Mortality and Cardiovascular Outcomes: A Systematic Review and Meta-Analysis

**DOI:** 10.3390/jcm11133799

**Published:** 2022-06-30

**Authors:** Vikash Jaiswal, Song Peng Ang, Kriti Lnu, Angela Ishak, Nishan Babu Pokhrel, Jia Ee Chia, Adrija Hajra, Monodeep Biswas, Andrija Matetic, Ravinder Dhatt, Mamas A. Mamas

**Affiliations:** 1 Department of Medicine, Larkin Community Hospital, South Miami, FL 33143, USA; vikash29jaxy@gmail.com; 2 Department of Internal Medicine, Rutgers Health/Community Medical Center, Toms River, NJ 08755, USA; hestonang23@gmail.com; 3 Department of Internal Medicine, UPMC Harrisburg, Harrisburg, PA 17105, USA; dhattrs@upmc.edu; 4 School of Medicine, European University Cyprus, Nicosia 2404, Cyprus; angela.ishak.10@gmail.com; 5 Department of Internal Medicine, Norwalk Hospital, Norwalk, CT 06850, USA; nishanpokhrel1@iom.edu.np; 6 School of Medicine, International Medical University, Kuala Lumpur 57000, Malaysia; jiaee1223@gmail.com; 7 Department of Internal Medicine, Jacobi Medical Center/Albert Einstein College of Medicine, The Bronx, NY 10461, USA; dradrijahajra@gmail.com; 8 Division of Cardiology, Wellspan Cardiology, Lancaster, PA 17602, USA; mbiswasmd@gmail.com; 9 Department of Cardiology, University Hospital of Split, 21000 Split, Croatia; andrija.matetic@gmail.com; 10 Keele Cardiovascular Research Group, Centre for Prognosis Research, Keele University, Keele ST5 5BG, UK

**Keywords:** pneumococcal vaccine, 23 PV vaccine, myocardial infarction, stroke, mortality

## Abstract

Various studies have suggested the possible cardiovascular (CV) protective effects of the pneumococcal vaccine (PV). Therefore, we conducted a meta-analysis to assess the association between recipients of PV with mortality and CV outcomes among patients with and without established cardiovascular disease. We performed a systematic literature search in PubMed, Embase, and Scopus for studies evaluating the effect of PV on mortality and CV outcomes. A total of 15 studies with 347,444 patients were included in the meta-analysis: 111,784 patients received PV (32%) and 235,660 patients were in the unvaccinated group (68%). Recipients of PV were associated with decreased all-cause mortality (HR, 0.76 (95% CI: 0.66 to 0.87), *p* < 0.001). PV was associated with a decrease in the incidence of myocardial infarction (MI) (HR, 0.73 (95% CI: 0.56–0.96), *p* = 0.02), without significant reduction in CV mortality (HR, 0.87 (95% CI: 0.72–1.07), *p* = 0.18) and stroke (HR, 1.01 (95% CI: 0.93–1.10), *p* = 0.82). Our study found PV was associated with decreased risk of all-cause mortality and MI. Future RCTs will be necessary to confirm benefits associated with receipt of PV.

## 1. Introduction

Cardiovascular diseases (CVD) are the leading cause of death globally, accounting for an estimated 17.9 million deaths in 2019 [1]. Previous data suggested that preexisting chronic cardiac conditions may predispose to community acquired pneumonia (CAP). The odds of CAP and invasive pneumococcal disease (IPD) in patients with CVD are significantly increased [2,3]. Furthermore, patients with CAP have an increased risk of cardiovascular complications, such as heart failure, acute coronary syndrome, cardiac arrhythmias and strokes through multiple mechanisms [4,5]. Streptococcus pneumoniae is known to produce 100 types of biochemically and serologically distinct capsule types or serotypes. Antibodies against one serotype do not offer protection against another. Major adverse cardiovascular events, or MACE, are an essential factor for mortality in patients with CAP by streptococcus pneumoniae [6]. It is estimated that there is an increase of more than 14% of new-onset heart failure and more than 7% of myocardial infarctions following CAP [7]. Furthermore, a previous meta-analysis reported an 18% occurrence of cardiovascular complications following CAP [8].

Various pathogen-driven mechanisms of cardiac damage as a result of *S. pneumoniae* pneumonia have been described. These include invasion of myocardium with the formation of microlesions [9,10], electrophysiological abnormalities [9,11,12], cardiomyocytes necrosis in a pneumolysin-dependent manner [8,10], enlargement and instability of atherosclerotic plaques [13], necroptosis in cardiomyocytes and macrophages infiltrating the heart [10,12,14], platelet activation and antibiotic-induced heart scarring [9,15]. Though most of the complications arising from these mechanisms manifest acutely, an increased cardiovascular risk may persist for as long as 10 years following infection [16].

The pneumococcal vaccine (PV) has been shown to reduce both the severity of CAP and reduce the subsequent risk of mortality associated with CAP [17]. The U.S. Centers for Disease Prevention and Control (CDC) recommends vaccination against pneumococcal disease for all adults with cardiovascular disease [18]. Similar recommendations in favor of pneumococcal vaccination were also encouraged for adult patients with heart failure in the 2021 European Society of Cardiology (ESC) guidelines and American Heart Association (AHA) 2013 guidelines for heart failure diagnosis and treatment [19,20].

Since PV has been shown to be efficacious, their protective effects amongst the elderly and in patients with CVD have been studied [21,22]. Several non-randomized studies (prospective and retrospective cohort studies) have been conducted in recent years and have shown conflicting results in terms of their effects on CV outcomes and mortality [21,22,23]. In addition, patients with prior CV diseases are at a higher risk of future CV events following pneumococcal infections. Therefore, we aim to perform a systematic review and meta-analysis to evaluate whether receipt of the pneumococcal vaccine among adults with and without history of cardiovascular disease is associated with a decreased risk of mortality and cardiovascular events.

## 2. Materials and Methods

This meta-analysis was conducted and reported following the Cochrane and PRISMA (Preferred reporting items for systematic review and Meta-analysis) 2020 guidelines and performed according to established methods, as described previously [24,25]. In addition, the pre-specified study protocol has been registered in PROSPERO: CRD42021277943.

### 2.1. Search Strategy

We conducted a systematic literature search in PubMed, Embase, Cochrane Library, and Scopus using predefined MESH terms using “AND” and “OR”. The following search terms were used: “pneumococcal Vaccine” OR “pneumonia” AND “mortality” OR “cardiovascular disease” OR “myocardial infarction” OR “cerebrovascular”. The search was performed from inception until February 2022 without any restrictions on the language of the studies. All the studies were carefully screened and exported to the Endnote 2020 library [26]. A manual check was carried out to crosscheck for any remaining duplicates. Two reviewers (SPA and AJ) reviewed the papers based on title and abstract. A third author (VJ) arbitrated discrepancies regarding the inclusion of studies.

### 2.2. Outcomes

The primary outcome for this meta-analysis was all-cause mortality between vaccinated and placebo/unvaccinated groups. The secondary outcomes were occurrence of myocardial infarction, stroke, and cardiovascular mortality. The definitions of all outcomes are provided in Table S1.

### 2.3. Inclusion Criteria

1. Studies with patients of age ≥ 18.
2. Studies including intervention and comparison groups where the intervention group was considered as patients who receive the pneumococcal vaccine (PV), while the comparison group was patients that were either unvaccinated or received a placebo.
3. Studies should report on the desired outcomes, i.e., all-cause mortality, risk of MI, stroke, and cardiovascular (CV) mortality.
4. Eligible study designs included RCTs, prospective and retrospective cohort studies.

### 2.4. Exclusion Criteria

1. Animal studies, abstracts, editorials, commentaries, systematic reviews, single patient case studies, letters, and studies with insufficient data were excluded.
2. Studies where the pneumococcal and influenza vaccines were compared instead of pneumococcal alone with placebo or unvaccinated groups were also excluded.

### 2.5. Data Extraction and Quality Assessment

All the included articles were extracted for the following data: study type, author, country, number of patients in both groups, age, sex, history of influenza vaccination, follow-up period, comorbidities, risk factors, primary and secondary outcomes. Three investigators (SPA, JA, and AI) independently appraised the potential risk of bias for observational studies using the Newcastle–Ottawa scale for cohort studies (Table S2). We then classified studies into low, moderate, or high quality based on the scores after evaluation.

### 2.6. Statistical Analysis

The vaccinated group was defined as patients who have received any form of PV. In contrast, the comparison group was defined as patients who were either not vaccinated or had received a placebo during the study period. We pooled the effect sizes comparing these two groups based on our predefined primary and secondary outcomes and analyzed each outcome individually using the random-effects model and DerSimonian–Laird method to account for between-study variations [27].

The estimated effect size was reported as a point estimate and 95% confidence interval (CI). A two-tailed *p*-value of less than 0.05 is statistically significant. Considering potential confounding factors, maximally adjusted estimates, where available, were used in the analysis. Between-study heterogeneity was calculated using the I^2^ test, with I^2^ > 50% and >75% considered to be moderately and highly heterogeneous, respectively [28]. In addition, we performed subgroup analyses on all outcomes based on CV risk and follow-up period (≥3 years vs. <3 years).

The high CV risk group includes studies where at least 50% of patients have established atherosclerotic CV diseases (coronary artery diseases, cerebrovascular diseases, or peripheral vascular diseases), heart failure, end-stage chronic kidney disease with estimated GFR < 15 mL/min/1.73 m^2^ or requiring dialysis, and diabetes mellitus with target end-organ damage. The remaining population fell under low or moderate CV risk. We performed sensitivity analyses by using the leave-out-one method, where studies were removed one at a time, and compared each overall effect size obtained with initial results [29]. Furthermore, meta-regression was performed to look for sources of heterogeneity and assess potential study-level covariates on the outcomes. The variables were as follows: age, gender, and comorbidities. We conducted all statistical analyses using STATA (StataCorp, Lakeway, TX, USA, version 17.0) [30].

## 3. Results

### 3.1. Literature Search

**Figure 1:**
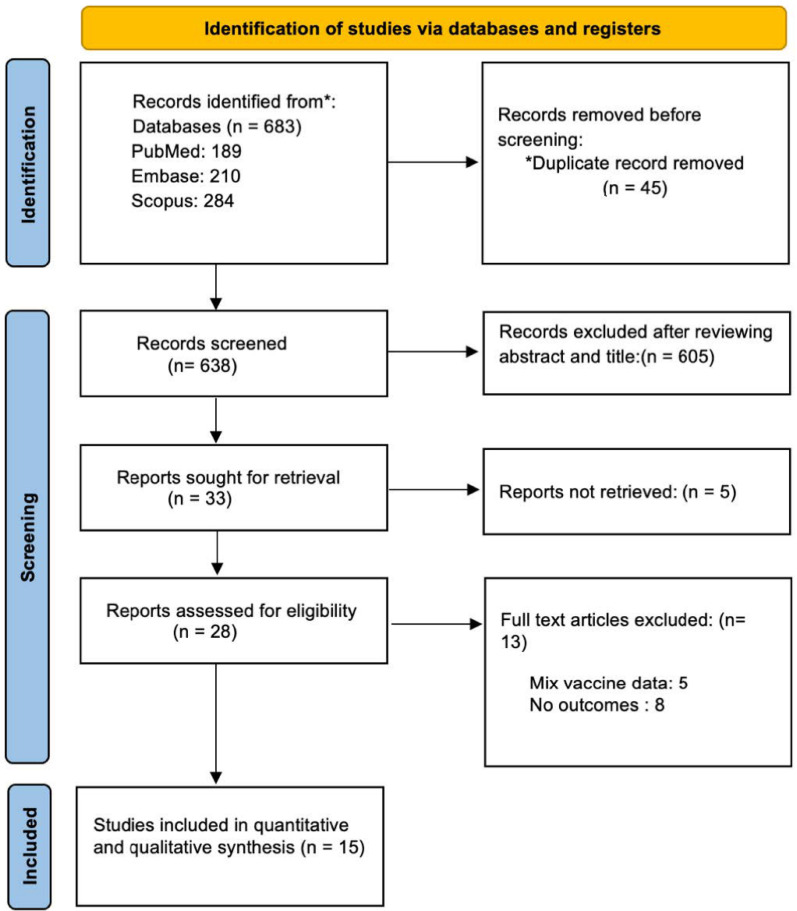
PRISMA flowchart of the search strategy for systematic review and meta-analysis.

A total of 683 articles were identified on the initial database screening by using the MESH terms. Forty-five duplicate articles were excluded manually based on author and titles of the study. A total of 605 articles were excluded based on outcomes, single-arm studies, mixed intervention groups, wrong drugs, and study designs that were not part of the inclusion criteria. Finally, 15 articles were included in the meta-analysis based on the eligibility criteria (Figure 1).

### 3.2. Studies Characteristics

A total of 347,444 patients were included in our analysis. The studies were undertaken in seven different countries with most being conducted in North America (seven in the USA and two in Canada), three in Europe (two in Spain and one in the United Kingdom) and three in Asia (China, Japan and Taiwan). The follow-up period varied among the included studies with a range from 3 months to 13 years. Table 1 summarizes the characteristics of the included studies.

The studies were performed in different patient population groups: five studies included patients that were at risk of developing or had a history of a MI or cerebrovascular accident, two studies included patients with or without a history of myocardial infarction (MI) or coronary artery disease (CAD), four studies included patients receiving hemodialysis, two studies included with chronic illness (including ischemic heart disease), and one study included all adults hospitalized for pneumonia while another included a cohort of men aged 45–69. In two studies, the patient population was divided into patients with high risk factors for CVD and those with low risk factors, while another included patients over >80 as one group and those 60–79 as another. Two studies used the same patient population but reported different outcomes, so they were included in the analysis.

### 3.3. Baseline Patient Demographics

A total of 111,784 patients received PV (32%) and 235,660 patients received a placebo or were unvaccinated (68%); 33% of all patients were male (25% in the PV while 36% in the unvaccinated group). The three most prevalent comorbidities were hypertension (29.5%; 29% in both groups separately), diabetes mellitus (29%; 23.9% in the PV group and 29.3% in the unvaccinated group) and hyperlipidemia (13.6%; 18% in the PV group and 10% in the unvaccinated group). The prevalence of smoking (either current or past) was also high in both groups: 24.1% in the PV group and 15.5% in the unvaccinated group. Table 2 describes the baseline characteristics of all included patients in the control and intervention group.

### 3.4. Meta-Analysis of Study Outcomes

#### 3.4.1. Primary Outcomes—All-Cause Mortality

**Figure 2:**
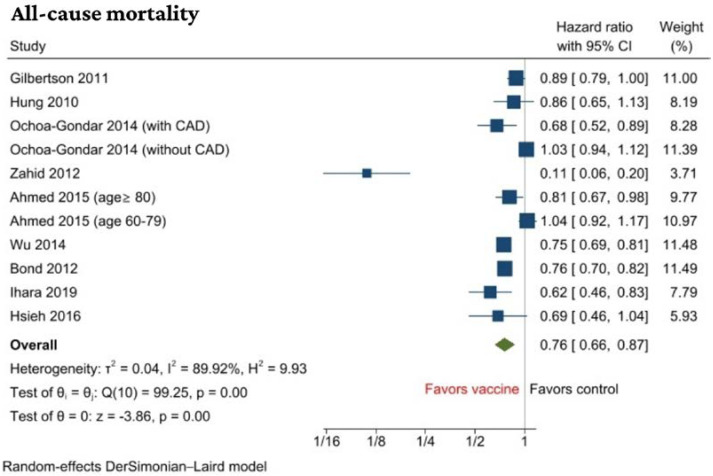
Forest plot of primary outcome: all-cause mortality [31,32,34,35,37,38,41,42,43].

Nine studies with a total of 209,607 patients with or without a history of CVD reported results of all-cause mortality [31,32,34,35,37,38,41,42,43]. PV was associated with a significant decrease in all-cause mortality when compared to no vaccination with a hazard ratio of (HR, 0.76 (95% CI: 0.66 to 0.87), *p* < 0.001, I^2^ = 90%) (Figure 2).

We performed a subgroup analysis based on CV risk stratification. It was found that the benefit of reduction in all-cause mortality was only in the high cardiovascular group (HR, 0.66 (95% CI: 0.56 to 0.79)) compared with low-to-moderate cardiovascular risk (HR, 0.96 (95% CI: 0.86 to 1.08)) and the subgroup difference was statistically significant (*p* < 0.01). The significant association seen in the total cohort seems to be due to weightage of the high CV risk cohort. Based on the subgroup analysis, only vaccinating high CV risk cohorts seem to have mortality benefit (Figure S1). There was no subgroup difference (*p* = 0.15) in terms of follow-up duration using 3 years as a cut-off period (Figure S2). Sensitivity analysis by the leave-one-out method showed that the odds of all-cause mortality remained significantly reduced in the vaccinated group compared to the control group, confirming the robustness of the results (Figure S3).

#### 3.4.2. Secondary Outcomes—Cardiovascular Mortality, Acute MI, and Stroke

**Figure 3:**
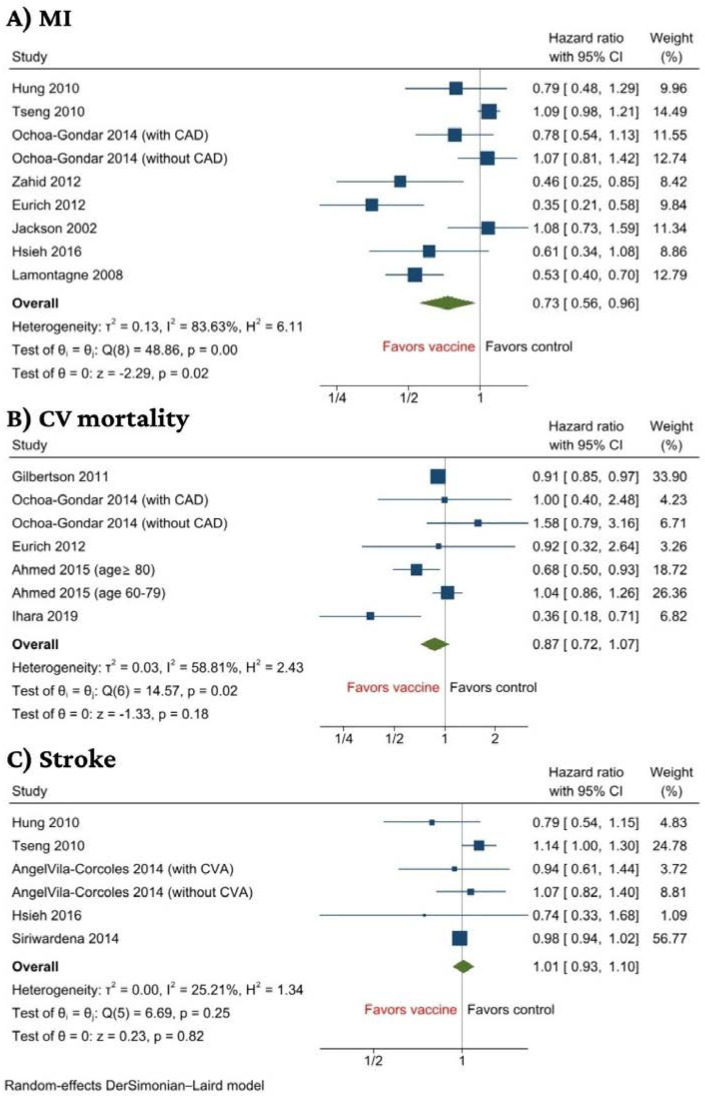
Forest plot of secondary outcomes including (**A**) MI, (**B**) CV mortality, and (**C**) Stroke [31,32,33,34,35,36,37,39,40,42,43,44,45].

Eight studies reported on acute MI with a total of 193,940 patients [32,33,34,35,36,40,43,45]. PV was associated with a significant decreased incidence of MI when compared to no vaccination with a hazard ratio of (HR, 0.73 (95% CI: 0.56–0.96), *p* = 0.02, I^2^ = 84%) (Figure 3A).

We performed a subgroup analysis based on CV risk stratification. Although there was no statistically significant subgroup difference between high cardiovascular risk against patients with low to moderate cardiovascular risk, the risk reduction effect of vaccination was lost when stratified. In the forest plot, it is evident that the association is statistically insignificant for the high CV risk cohort and low to moderate risk cohort; however, it is significant in the total cohort, suggesting the studies in each subgroup may be inadequate and thus lack statistical power to detect a significant beneficial effect of vaccination (Figure S4). We found a significant subgroup difference based on follow-up duration (*p* = 0.04), with the reduction in acute MI being more profound in studies with a follow-up duration of less than 3 years (HR 0.60, 95% CI 0.41 to 0.89) compared to that of 3 years or more (HR 0.96, 95% CI 0.78 to 1.18) (Figure S5). Sensitivity analysis via successive exclusion of each individual study showed that the results, in general, remained significant, except upon removal of studies by Zahid et al., Lamontagne et al. or Eurich et al., suggesting that results might be influenced by these studies (Figure S6).

There were no statistical differences between PV status and the rest of the secondary outcomes (CV morality and stroke). Five studies reported on CV mortality with 157,708 patients [31,34,37,40,42]. The analysis revealed no significant association between receipt of PV and CV mortality (HR, 0.87 (95% CI: 0.72–1.07), *p* = 0.18, I^2^ = 58.8%) (Figure 3B). Finally, five studies reported on stroke with 179,649 patients [32,33,36,43,44]. The analysis revealed no significant association between recipients of PV and stroke (HR, 1.01 (95% CI: 0.93–1.10), *p* = 0.82, I^2^ = 25.2%) (Figure 3C). Results of sensitivity analysis on CV mortality and stroke were in concordance with the main findings (Figure S7A,B).

#### 3.4.3. Meta-Regression

Meta-regression was performed for the outcomes of all-cause mortality and acute MI to explore for underlying effect modifiers. The following variables were considered: age, sex (male), specific comorbidities (HTN, DM, hyperlipidemia, prior MI or CVA, chronic kidney disease), smoking, and prior influenza vaccine. The results from the meta-regression are summarized in Table 3.

Meta-regression revealed male sex (coefficient −0.011, *p* = 0.027) (Figure S8) as the sole significant negative predictor of the observed effect size on all-cause mortality.

#### 3.4.4. Publication Bias

Publication bias was assessed using a funnel plot and Egger’s regression test. Possible publication bias was detected through funnel plot asymmetry and Egger’s test (*p* < 0.05) for all-cause mortality (Figure S9). Overall estimates remain similar, with the vaccinated group showing a greater reduction in all-cause mortality after trim-and-fill analysis with the addition of three studies (HR, 0.85 (95% CI 0.74 to 0.99), *p* = 0.04). Otherwise, publication bias was not detected for the other outcomes including cardiovascular mortality (*p* = 0.76), MI (*p* = 0.09), and stroke (*p* = 0.45) (Figure S10A–C).

## 4. Discussion

To the best of our knowledge, this is the most comprehensive and the largest meta-analysis, consisting of 15 studies with 347,444 participants. The participants were followed for a mean period of 2.89 ± 3.25 years. Pooled results demonstrated that receipt of PV was associated with a 24% decrease in all-cause mortality and a 27% decrease in the risk of MI when compared to the unvaccinated group, with no significant interaction by baseline cardiovascular risk. Our findings support the recommendations of the Centers for Disease Prevention and Control (CDC) for vaccination against pneumococcal disease for all adults with cardiovascular disease and represent the best available current evidence.

The all-cause mortality benefit from PV in our study is in line with the findings of previous analyses. A previous meta-analysis revealed that there was a significant reduction in all-cause mortality in patients receiving PV; however, their analysis only considered patients with established CV disease (such as heart failure) or those with a very high CV risk [46]. On the other hand, our study demonstrated benefits in adult patients, regardless of the baseline cardiovascular risk. In contrast to previous meta-analyses, our study failed to show a protection effect on CV mortality after PV [47,48]. While most studies to date, including ours, showed no benefits in terms of stroke, a meta-analysis conducted by Vlachopoulos et al. revealed a decreased risk of stroke only in elderly populations [46,47,48].

With the currently available evidence, the cardioprotection offered by the PV could be associated with inhibition of pneumococcal infections and decreased inflammation or due to immunomodulatory mechanisms [49]. *S. pneumoniae* has been demonstrated to activate platelets, invade the myocardium, and cause cardiomyocyte necrosis, resulting in instability of the atherosclerotic plaques [23,49,50,51]. Additionally, systemic inflammation during pneumococcal infections promotes a thrombogenic state and increases the metabolic demand of oxygen [52]. With the mismatch in supply and demand of oxygen, and the propensity towards rupture of extremely thin-capped fibroatheroma, the risk of both Type 1 and Type 2 MI is increased [50,53,54].

The immunomodulatory mechanism of PV may also be associated with suppression of atherosclerosis development/progression. It is based on the “antigen mimicry” between oxidized LDL (oxLDL), a component of atherosclerotic plaques, and phosphorylcholine lipid antigens expressed on the cell wall polysaccharide of *Streptococcus pneumoniae* [55,56]. Previous studies have reported a 40% reduction in atherosclerotic plaque in an animal model after pneumococcal vaccination [51]. Antibodies produced against *S. pneumoniae* after PV also bind to oxidized phospholipid epitopes on oxLDL and prevent uptake of oxLDL by macrophages, thus causing decreased progression of atherogenesis in pneumococcal-immunized mice [55,56]. These findings suggest that PV exerts beneficial effects by directly preventing CV events via the immunomodulatory mechanism. Therefore, it may be argued that current AHA and ESC recommendations for PV could be expanded for all patients with CVD risk factors and not just patients with heart failure [19,20], although this would require further prospective studies.

The protective effect of PV appears to be associated with follow-up duration. In a meta-analysis of cohort studies, the protective role of PV was found to be diminished over time, where PV offered higher protective value during shorter follow-up periods when compared to longer follow-up [48]. Similarly, our study showed that a shorter follow-up duration is associated with a larger magnitude of reduction in the incidence of MI in the vaccinated group, suggesting that the cardioprotective effect may wane over time.

### Strengths and Limitations

We have used an all-inclusive search strategy to explore all the relevant studies comparing pneumococcal vaccine with placebo/control to date. Our analysis is the most up-to-date and included the largest sample size to check the real-world data and analyzed PV’s effect on all adult populations. A major limitation is that the included studies were observational in nature, and thus, may be affected by other confounding factors which were not possible to account for. While our results had high heterogeneity, results after sensitivity analysis by leave-one-out analysis and exclusion of studies through guidance of Galbraith plot, in general, remained significant and in line with the main analysis. Whilst there are no RCTs, a multi-center phase 3 RCT with a sample of 4750 participants aged 55–60 years having two or more CVD risk factors but no known CVD events is ongoing (ACTRN12615000536561) [57].

In a study by Africano et al., MACE were independently associated with disease caused by serotypes 3 and 9n. The serotype 3 capsule is included in the current 13-valent conjugate vaccine (PCV13) formulation and pneumococcal polysaccharide vaccine (PPSV23). Still, it results in severe disease and causes MACE. Another serotype, 9N, is associated with invasive pneumonia and severe disease. The authors also found that patients infected with serotype 9n were more susceptible to MACE during invasive pneumonia. Serotype 9n is not covered by the currently available vaccine and was associated with mortality along with serotype 3 [6]. However, we did not have enough data to compare different types of vaccines against different serotypes. Information regarding different pneumococcal vaccines was limited; therefore, we were not able to analyze the outcomes by vaccine type. Lastly, limited or lack of data availability on certain endpoints such as MACE, stroke, cardiovascular mortality may have affected our assessment on these outcomes.

## 5. Conclusions

Pneumococcal infections in patients with cardiovascular disease are associated with an increased morbidity and mortality. The findings of this meta-analysis suggest that PV could prove to be cardioprotective as evidenced by the reduction in risk of MI and all-cause mortality. However, future randomized controlled trials are required to demonstrate the evidence suggested by our meta-analysis to inform future guidelines, and vaccination programs can be implemented in those with the greatest benefit.

## Figures and Tables

**Table 1:** Characteristics of included studies.

Author	Year	Study Design	Country	Study Population	Follow Up, Years	No of Patients, n (PV/Control)	Outcomes	Outcome Adjustment
Gilbertson et al. [31]	2011	prospective	USA	Hemodialysis patients	0.5	25,091/93,442	All-cause mortality, Cardiovascular mortality	Patient demographics, comorbidity, and influenza vaccinations
Hung et al. [32]	2010	prospective	Hong Kong	Patients aged ≥65 years and had ≥1 of the following chronic illness: asthma, chronic obstructive pulmonary disease (COPD), coronary artery disease, hypertension, diabetes mellitus, stroke, chronic renal or liver disease, or malignancy.	1.25	1875/25,393	All-cause mortality, Stroke, Acute MI	Sex and COPD
Tseng et al. [33]	2010	prospective	USA	Men aged 45 to 69	4.7	36,309/47,861	Incidence of Stroke and Acute MI	Age, region, race/ethnicity, smoking, BMI, physical inactivity, income, education, history of MI, history of stroke, history of PAD, high cholesterol, high BP, DM, other HD, nutrition, alcohol consumption, outpatient visits, sedentary status, influenza vaccinations
Ochoa-Gondar et al. [34]	2014	prospective	Spain	Adults aged 60 years old and older, with or without prior h/o CAD	3	8981/18,223	All-cause mortality, death from MI	Age, sex, influenza vaccination status, number of outpatient visits in previous 12-month, nursing-home residence, history of pneumonia, cerebrovascular disease, chronic pulmonary disease, chronic heart disease, chronic liver disease, chronic nephropathy, DM, cancer, dementia, immunodeficiency, HTN, hypercholesterolemia, obesity, alcoholism, smoking, and immunosuppressive medication
Zahid et al. [35]	2012	retrospective	USA	Patients with suspected ACS	0.5	507/579	All-cause mortality, acute MI	Propensity score for pneumococcal vaccination. Adjusted for variables including influenza vaccination only, pneumococcal and influenza vaccinations, age (per year), SBP < 90 mmHg, pulmonary edema on admission, hemoglobin < 11.5 gm/dL, left ventricular ejection fraction < 35%, smoking (past/current), increased troponin, DM, statins, and missing data
Eurich et al. [36]	2012	prospective	Canada	Patients aged >17 years with pneumonia	0.25	725/5446	Fatal and non-fatal ACS	Pneumonia severity based on the PSI; comorbidities including COPD, DM, CAD, functional status, smoking status and CV and other medications
Ahmed et al. [37]	2015	prospective	USA	Community dwelling adults aged 65 and above, with h/o MI or coronary heart disease	13	1424/3866	All-cause mortality, cardiovascular mortality	Age ≥ 85 years, sex, race, married, education college or higher, income ≥ USD 25 K, smoking ≥32 pack years, walk blocks last week ≥ 10, body mass index ≥ 25 kg/m^2^, instrumental activities of daily living ≥1, Centers for Epidemiologic Studies Depression (CES-D) scale score, MMSE, influenza vaccination, CHD, HTN, DM, stroke, acute MI, AF, LVH, LV systolic dysfunction, LBBB, CKD, COPD, pneumonia, serum CRP ≥ 2.4 mg/L
Wu et al. [38]	2014	retrospective	USA	Adults with HF	1	7108/586	All-cause mortality	Age; sex; race; hospital days last 6 months; number of hospitalizations prior 6 months; prior HF hospitalization; Elixhauser risk index, prior MI; fiscal year of the assessment; hematocrit, MABP, pulse, creatinine clearance, and clustering within hospitals
Vila-Corcoles et al. [39]	2014	prospective	Spain	Adults aged 60 years old and older, with or without prior CVA	3	8981/1823	All-cause mortality	Age, sex, influenza vaccination status, number of outpatient visits in previous 12 months, nursing-home residence, history of pneumonia, coronary artery disease, cerebrovascular disease, chronic pulmonary disease, chronic heart disease, chronic liver disease, chronic nephropathy, DM, cancer, immunodeficiency, dementia, HTN, hypercholesterolemia, obesity, alcoholism, smoking, and immunosuppressive medication
Jackson et al. [40]	2002	retrospective	USA	Patients with a first nonfatal myocardial infarction	2.3	661/1378	Acute MI	Age, sex, shock, or severe CHF (defined as requiring hemodynamic monitoring and/or vasopressor support) during hospitalization for the incident myocardial infarction, smoking status, DM, HTN, chronic CHF, COPD/asthma, and cardiac medication use
Bond et al. [41]	2012	retrospective	USA	Dialysis patients	1	1297/20,180	All-cause mortality	Age, race, sex, time on dialysis (vintage), modality (hemodialysis, continuous cyclic peritoneal dialysis, or continuous ambulatory peritoneal dialysis), diabetes as primary cause of ESRD (yes or no), comorbid conditions at dialysis therapy initiation (congestive heart failure, cerebrovascular disease, peripheral vascular disease, history of hypertension, chronic obstructive pulmonary disease, and malignant neoplasm), and mean monthly patient laboratory values for albumin, hemoglobin, and Kt/V during the 3-month influenza vaccination period
Ihara et al. [42]	2019	retrospective	Japan	Dialysis patients	5	255/255	All-cause mortality, CV mortality	Propensity score-matched using variables including age, sex, body mass index (BMI), duration of dialysis, serum level of albumin, influenza vaccination in 2010, history of arteriosclerotic heart disease, chronic heart failure, peripheral vascular disease, and diabetes mellitus (DM)
Hsieh et al. [43]	2016	retrospective	Taiwan	Patients aged >50 years with chronic renal failure on maintenance hemodialysis	5	168/377	All-cause mortality, acute MI, stroke	-
Siriwardena et al. [44]	2014	case–control	UK	Adults (>40) with a first diagnosis of MI	1	26,847/13,615	Stroke	Asthma, COPD, or CAD, stroke or TIA, DM, hyperlipidemia, splenectomy, chronic liver disease, CRF, immunosuppression, HIV/AIDS, family history of AMI, PVD, HTN, smoking status, treatment with acetylsalicylic acid or statins, or antihypertensives, GP consultations, BMI
Lamontagne et al. [45]	2008	case–control	Canada	Patients at risk of MI	1.8	536/4459	Acute MI	Matched for age, sex and hospitalization index date. Comparisons were adjusted for COPD, CRF, DM, previous *S. pneumoniae* infection, splenectomy

**Table 2:** Baseline patient demographics and comorbidities *.

Variables	Gilbertson 2011 [31]	Hung 2010 [32]	Tseng 2010 [33]	Ochoa-Gondar 2014 [34]	Zahid 2012 [35]	Eurich 2012 [36]	Ahmed 2015 [37]	Wu 2014 [38]	Vila-Corcoles 2014 [39]	Jackson 2002 [40]	Bond 2012 [41]	Ihara 2019 [42]	Hsieh 2016 [43]	Siriwardena 2014 [44]	Lamontagne 2008 [45]
Sample (n) PV/Placebo	25,091/93,442	1875/25,393	36,309/47,861	8981/18,223	507/579	725/5446	1424/3866	7108/586	8981/18,223	661/1378	1297/20,180	255/255	168/377	26,847/13,615	536/4459
Age, years (Mean)			/55.2	-	68.9/			71.82/64.55	-	-	59.3/59.8	61.3/62	-	-	-
Male, %	52.3/53.2	45/47	-	45.2/44	97.8/97.4	48/53	41.57/42.03	98.2/98.8	45.2/44.3	-	48.7/52.0	68.2/67.5	-	-	-
Obesity	-	-	27.99/24.68	37.3/17.3	-	-	-	-	35.1/27.9	-	-	-	-	38.3/54.5	-
Past Influenza Vaccination, %	89.6/71	-		82.1/38.4	69/100	90/3	77.67/31.14	-	82.1/38.4	-	-/70.3	78.4/74.9	-	0/0	-
Comorbidities															
HTN, %	29/30.3	59.8/60.7	45.23/30.3	59.1/50.7	73.4/65.5	-	60.11/57.55	-	59.1/50.7	-	78.9/80.1	-	-	50.3/26.2	-
HLD, %	-	-	46.53/35.8	40.2/34.9	-	-	-	-	40.2/34.9	-	-	-	-	13.4/6.6	-
DM, %	62.1/59.4	24.5/24.1	20.57/6.2	24.4/20.4	41.8/36.96	90/3	15.03/15.7	-	24.4/20.4	-	24.1/22.8	60/61.2	-	16/4.2	-
Smoker, %	-	14.8/13.5	62.78/53.31	35.1/29.5	67.7/72.9	52.69/33.29	-	-	35.1/29.46	-	7.3/5.9	-	-	62.3/38.9	-
COPD, %	26/24.1	3.9/2	-	8.26/7.8	-	39/15	19.6/9.7	-	8.26/7.8	-	4.3/3.9	-	-	23.4/7.8	-
CHF, %	51.1/49.8	8.7/7.9	3.9/1.6	14.7/11.4	18/15.9	-	7.4/7.2	24.6/20.3		-	22.4/22.0	23.9/26.7	-	-	-
Chronic Liver Disease, %	12.4/13.3	0.3/0.3	-	2.66/2.1	-	-	-	-	2.66/2.1	-	-	2/5.5	-	0.5/0.2	-
Previous MI, %	-	1/1.2	-	-	24.9/21.76	-	8.15/7.79	34.8/24.4	-	-	-	-	-	-	-
Previous Stroke, %	24.3/23	0.39/7.1	-	5.4/4.43	14.8/9.67	-	4.78/3.52	-	5.4/4.43	-	-	20.4/23.5	-	-	-
Kidney Disease, %	100/100	2.6/2.3	-	2.38/2.44	16.8/16.75	13/7	-	-	2.38/2.4	-	100/100	100/100	100/100	12.7/2.7	-

* Table 2 data are arranged in PV/Placebo format.

**Table 3:** Meta-regression of potential effect modifiers for all study outcomes.

Meta-Regression Variables	All-Cause Mortality		MI	
	Coefficient	*p* *	Coefficient	*p* *
Demographics				
Age	0.017	0.267	0.009	0.814
Male	−0.011	0.027	−0.009	0.466
Comorbidities				
HTN	−0.002	0.727	-	-
DM	−0.004	0.432	0.005	0.758
HLD	-	-	-	-
Prior CVA	−0.008	0.589	-	-
Prior MI	-	-	-	-
HF	0.002	0.765		
CVD	−0.011	0.161	-	-
COPD	0.007	0.490	-	-
CKD	0.001	0.593	−0.001	0.806
Smoking	-	-	−0.002	0.876
Prior Influenza vaccine	0.011	0.198	-	-

* denotes significant results (*p* < 0.05).

## Data Availability

The data presented in this study are available in the article and Supplementary Material.

## Supplementary Materials

The following supporting information can be downloaded at: https://www.mdpi.com/article/10.3390/jcm11133799/s1, Table S1: Definitions of outcomes; Table S2: Newcastle–Ottawa scale for quality assessment and bias assessment of observational studies; Figure S1: Subgroup analyses based on cardiovascular risk for primary outcome: all-cause mortality; Figure S2: Subgroup analyses based on follow-up duration for primary outcome: all-cause mortality; Figure S3: Leave-one-out analysis for primary outcome: all-cause mortality; Figure S4: Subgroup analyses based on cardiovascular risk for secondary outcome: MI; Figure S5: Subgroup analyses based on follow-up duration for secondary outcome: MI; Figure S6: Leave-one-out analysis for secondary outcome: MI; Figure S7: Leave-one-out analysis for secondary outcome: (A) CV mortality (B) Stroke; Figure S8: Meta-regression of male as an effect modifier for all-cause mortality; Figure S9: Funnel plots for publication bias for primary outcome: all-cause mortality; Figure S10: Funnel plots after trim-and-fill for primary outcome: all-cause mortality.
