# Supplementary material for: Effect of Pneumococcal Vaccine on Mortality and Cardiovascular Outcomes: A Systematic Review and Meta-Analysis
Source: J Clin Med. 2022 Jun 30;11(13):3799. doi: 10.3390/jcm11133799 (PMC9267914; doi:10.3390/jcm11133799)
Supplement: Supplementary file 1 [file jcm-11-03799-s001.zip › jcm-1757613-supplementary.pdf]

Supplementary figures and tables legend

Table S1: Definition of Outcomes

Table S2: Newcastle-Ottawa scale for quality assessment and bias assessment of observational studies

Figure S1: Subgroup analyses based on cardiovascular risk for primary outcome: all-cause mortality

Figure S2: Subgroup analyses based on follow-up duration for primary outcome: all-cause mortality

Figure S3: Leave-one-out analysis for primary outcome: all-cause mortality

Figure S4: Subgroup analyses based on cardiovascular risk for secondary outcome: MI

Figure S5: Subgroup analyses based on follow-up duration for secondary outcome: MI

Figure S6: Leave-one-out analysis for secondary outcome: MI

Figure S7: Leave-one-out analysis for secondary outcome: (A) CV mortality (B) Stroke

Figure S8: Meta-regression of male as an effect modifier for all-cause mortality

Figure S9: Funnel plots for publication bias for primary outcome: all-cause mortality

Figure S10: Funnel plots after trim-and-fill for primary outcome: all-cause mortality

Supplementary File
Index

Study question:
Outcome of Pneumococcal vaccinated group and Placebo among patients with and without established cardiovascular disease

PICO Questionnaires
P= patient/population (Patients with and without established cardiovascular disease)
I= Intervention arm (Pneumococcal vaccinated group)
C= Comparison arm (Placebo/control group)
O= outcomes (primary outcome): all-cause mortality. Secondary outcomes: myocardial infarction, cardi mortality, and stroke.

Search Strategy:

(((Pneumococcal Vaccine) OR (PPV)) AND ((Cardiovascular outcomes) OR (Myocardial Infarction)) AND ((Heart failure)) AND ((Acute coronary syndrome)).

Table S1: Definition of Outcomes

Study Variable	Definition
**All Cause Mortality**	All studies that indicated mortality used a primary endpoint as all-cause mortality. All-cause mortality is defined as death from any cause that occured during follow-up period among participants in intervention and comparison groups.
**MACE**	Major adverse cardiovascular events (MACE) is defined as a composite of in-hospital all-cause death, acute myocardial infarction, or any cardiovascular events.
**Acute Myocardial Infarction**	Presumptive cases of AMI were initially identified on the basis of primary hospital discharge ICD-9 diagnosis codes for acute myocardial infarction (ICD-9 410).<br>AMI was defined as a detection of rise and/or fall in cardiac biomarkers together with at least one of the following: symptoms of ischaemia, ECG changes indicative of new ischaemia (new ST- T changes, new left bundle branch block and/or development of pathological Q waves) and/or imaging evidence of new loss of viable myocardium or new regional wall motion abnormality
**Stroke**	Stroke was defined as an accident which led to loss of neurological function with residual symptoms at least 24 hrs after onset, presumed to be from vascular etiology.
**Cardiovascular mortality**	Cardiovascular mortality is defined as death occurred due cardiovascular disease such as one of the following: acute myocardial infarction, acute rheumatic fever and chronic rheumatic heart disease, ischemic heart disease,

	all other form of heart disease, aortic aneurysm and dissection, atherosclerosis, disease of pericardium and myocarditis, hypertension and hypertensive renal disease, heart failure, and disorders of circulatory system.

Table S2: Newcastle-Ottawa scale for quality assessment and bias assessment of observational studies

Studies	Selection				Comparability	Outcomes			Total*
	Representatives of exposed group	Selection of control group	Exposure ascertainment	Outcomes not present at start		Outcome assessment	Adequacy of follow up duration	Adequacy of follow up of cohort	
Gilbertson 2011	★	★	★		★	★		★	6
Hung 2010	★	★	★		★	★	★		6
Tseng 2010	★	★	★	★	★★	★	★	★	9
Ochoa-Gondar 2014	★	★	★		★	★	★	★	7
Zahid 2012	★	★	★	★	★	★		★	7
Eurich 2012	★	★	★		★	★		★	6
Ahmed 2015	★	★	★	★	★	★	★	★	8
Wu 2014	★	★	★	★	★	★	★	★	8
AngelVila-Corcoles 2014	★	★	★		★	★	★	★	7
Jackson 2002	★	★	★	★	★★	★	★	★	9
Bond 2012	★	★	★	★	★	★	★		7

Ihara 2019	★	★	★	★	★★	★	★		8
Hsieh 2016	★	★	★		★★	★	★		7
Siriwardena 2014	★	★	★	★	★	★	★		7
Lamontagne 2008	★	★	★	★	★	★	★		7

*Score >6 was considered as an adequate quality study.

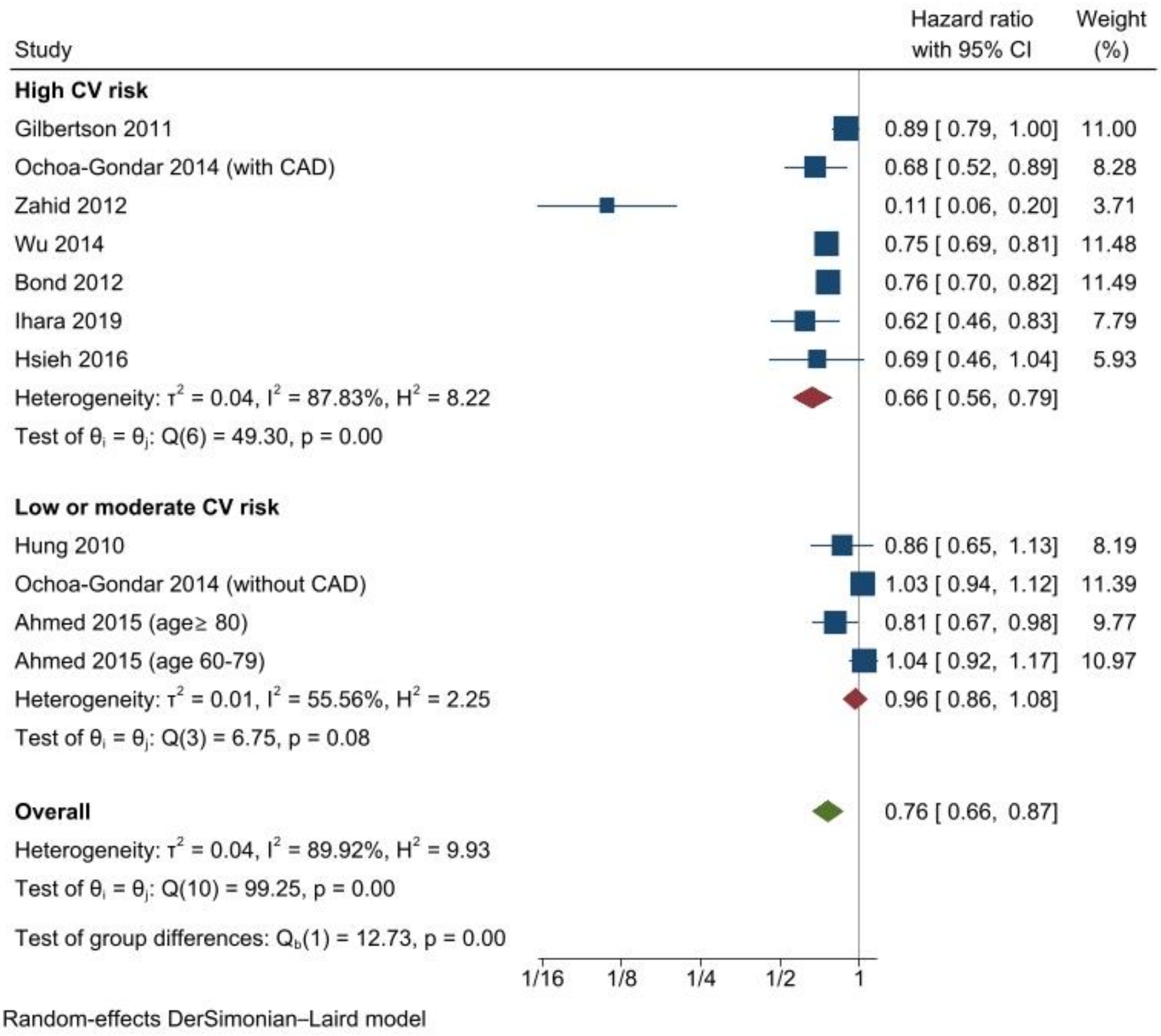

Figure S1: Subgroup analyses based on cardiovascular risk for primary outcome: all-cause mortality

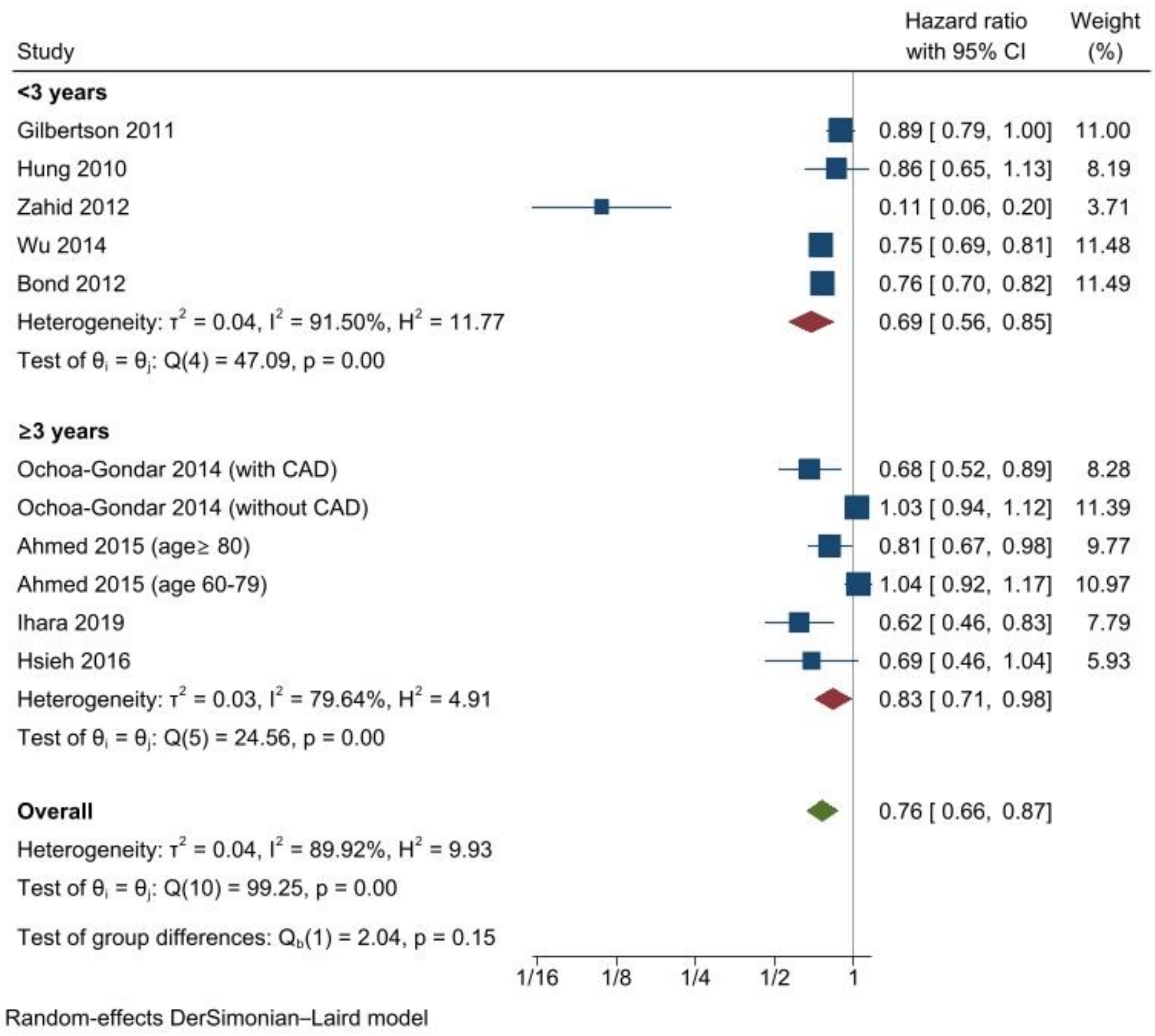

Figure S2: Subgroup analyses based on follow-up duration for primary outcome: all-cause mortality

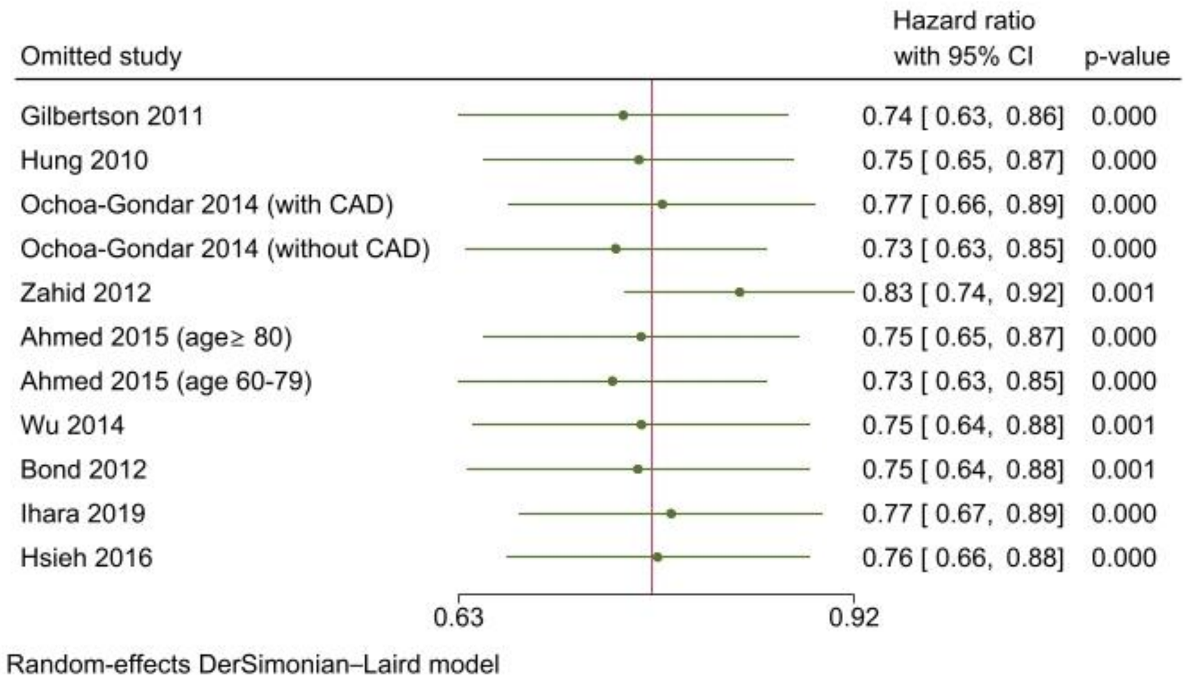

Figure S3: Leave-one-out analysis for primary outcome: all-cause mortality

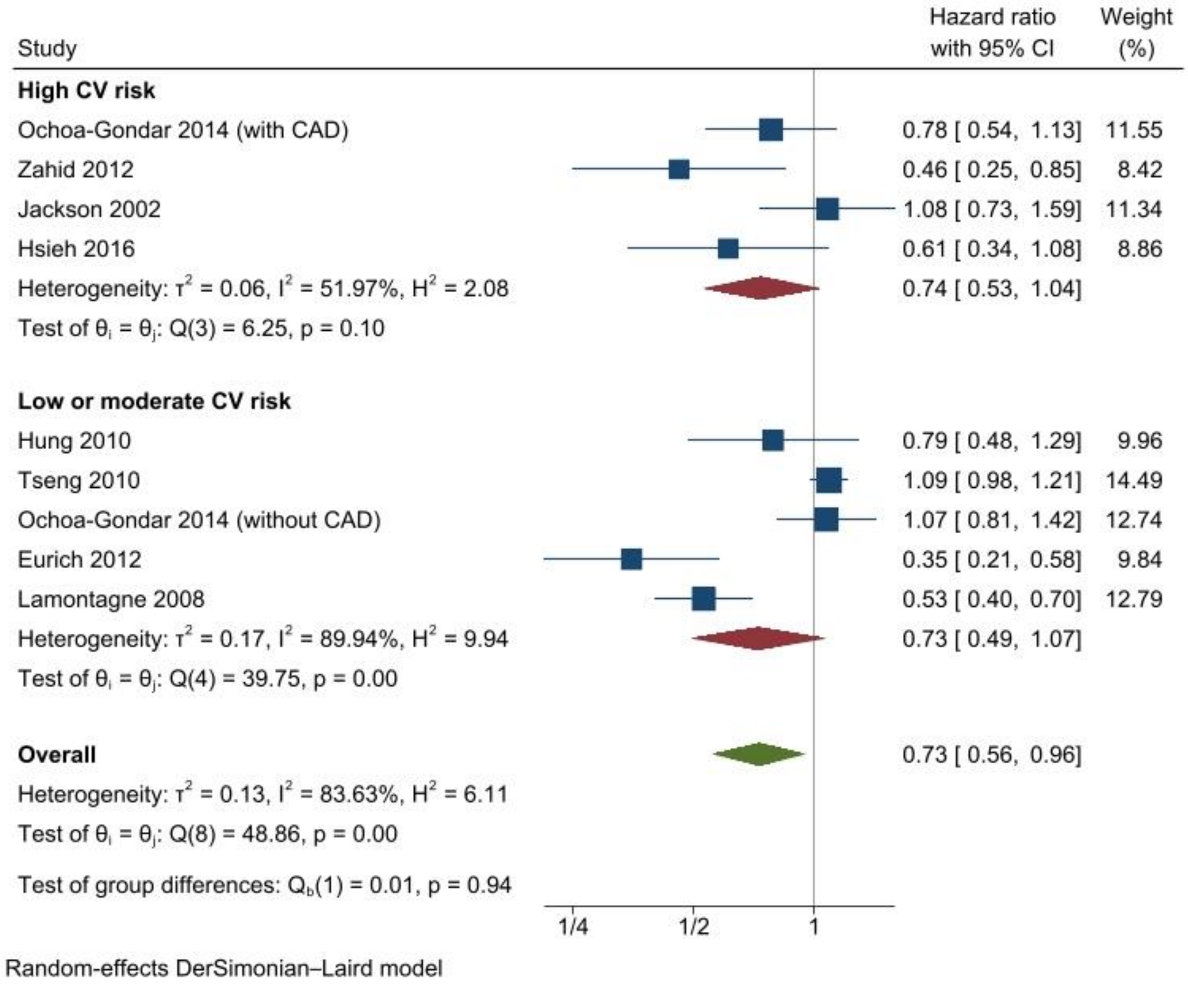

Figure S4: Subgroup analyses based on cardiovascular risk for secondary outcome: MI

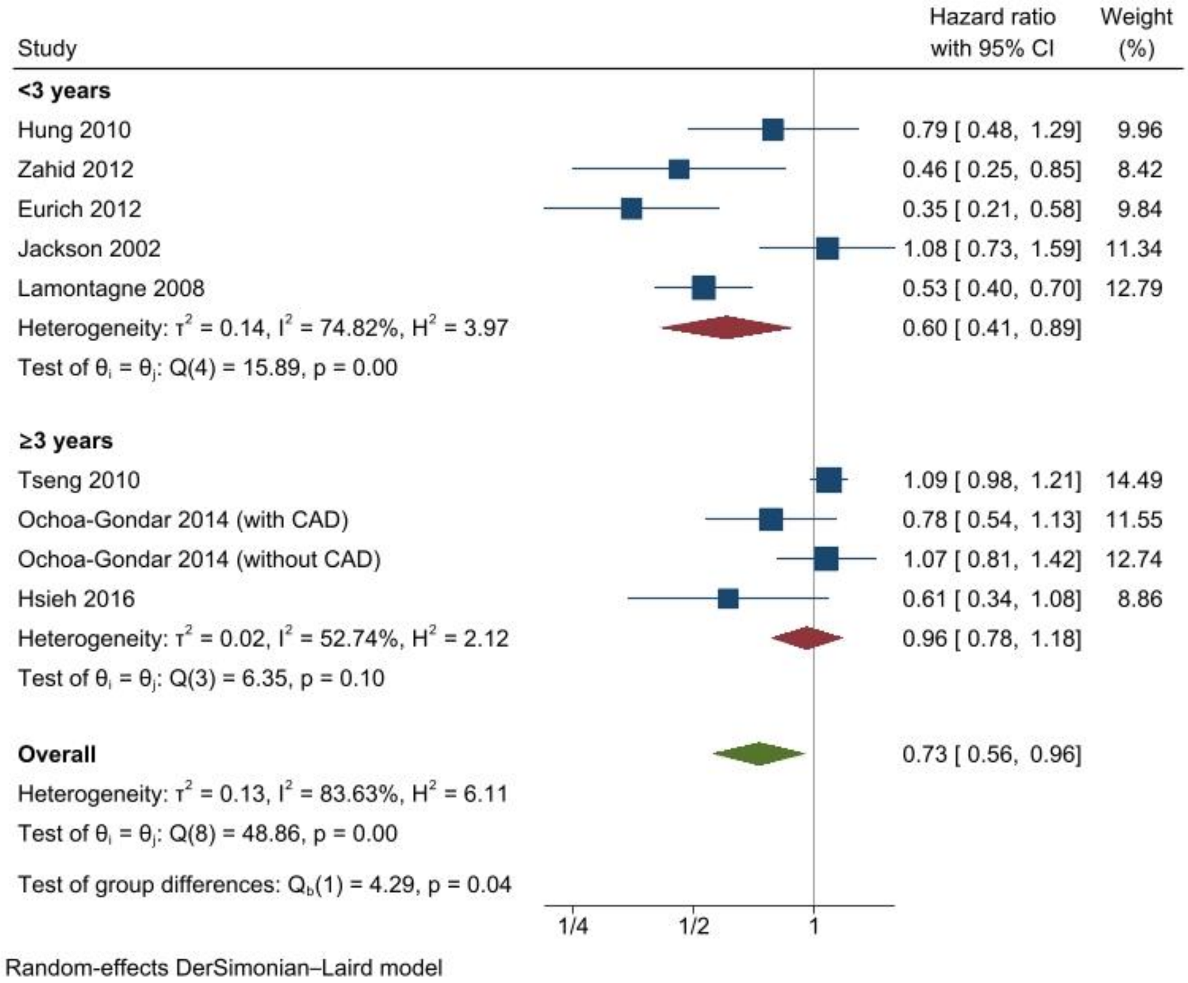

Figure S5: Subgroup analyses based on follow-up duration for secondary outcome: MI

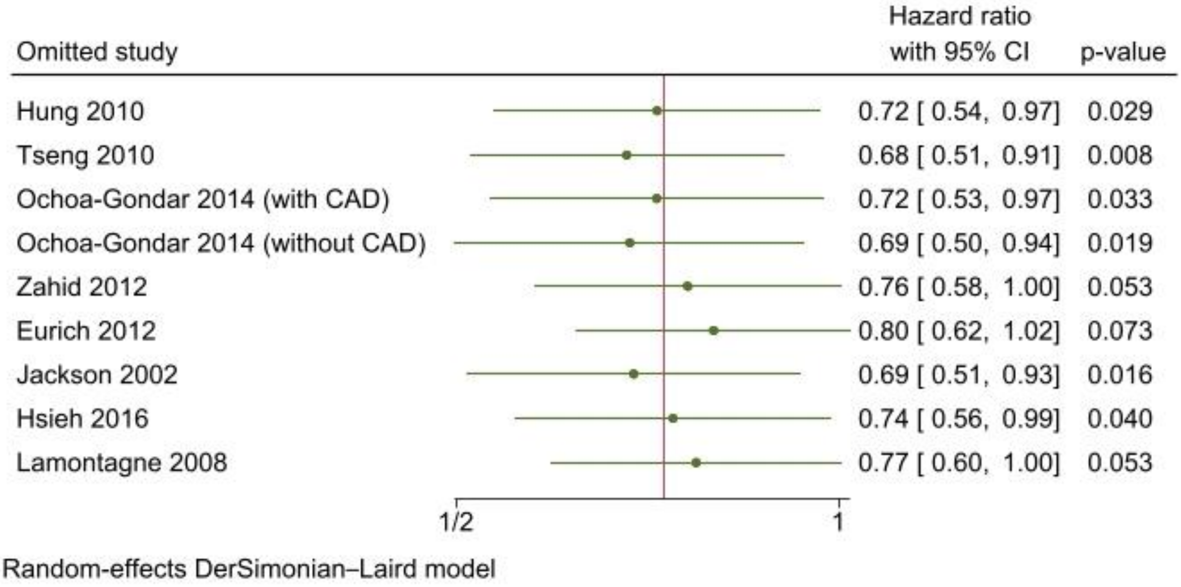

Figure S6: Leave-one-out analysis for secondary outcome: MI

A) CV mortality

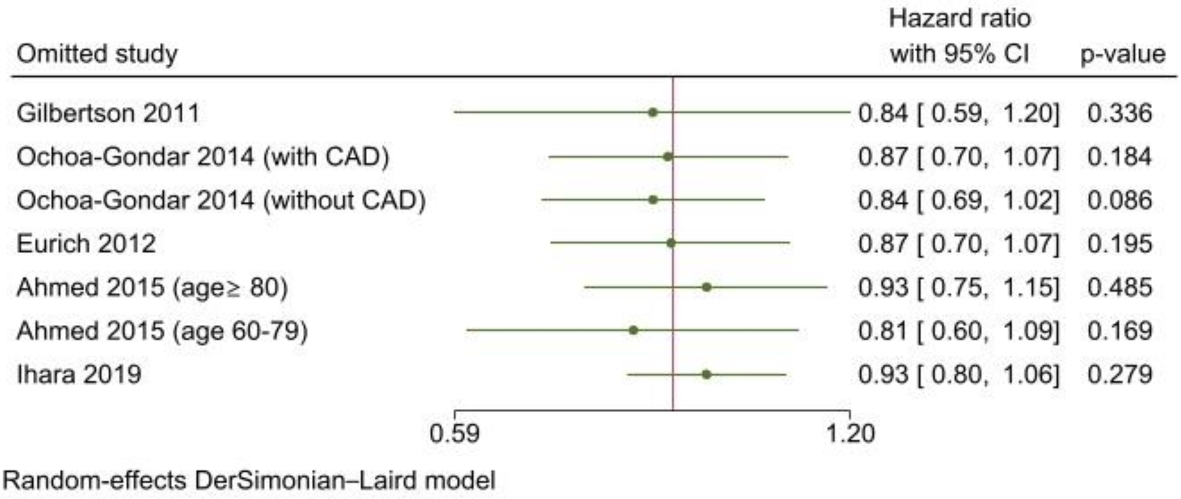

B) Stroke

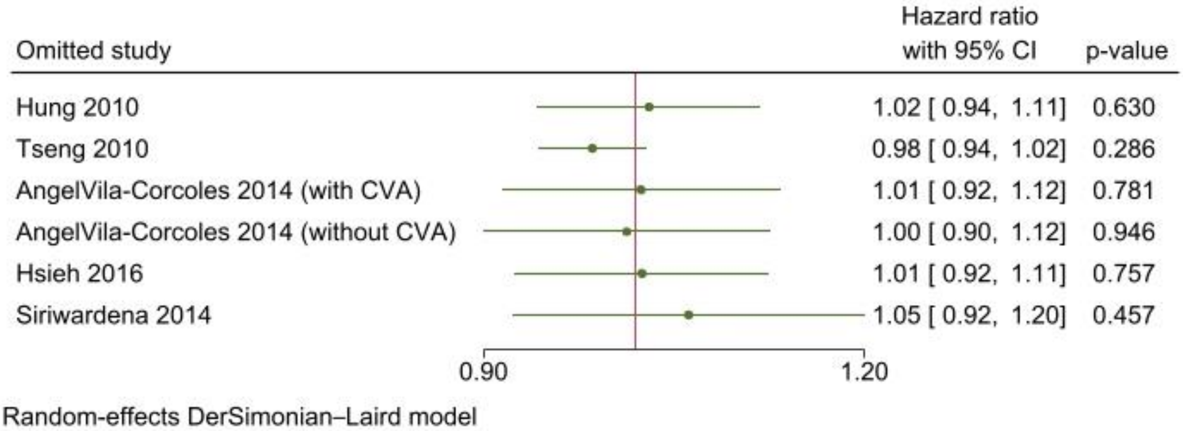

Figure S7: Leave-one-out analysis for secondary outcome: (A) CV mortality (B) Stroke

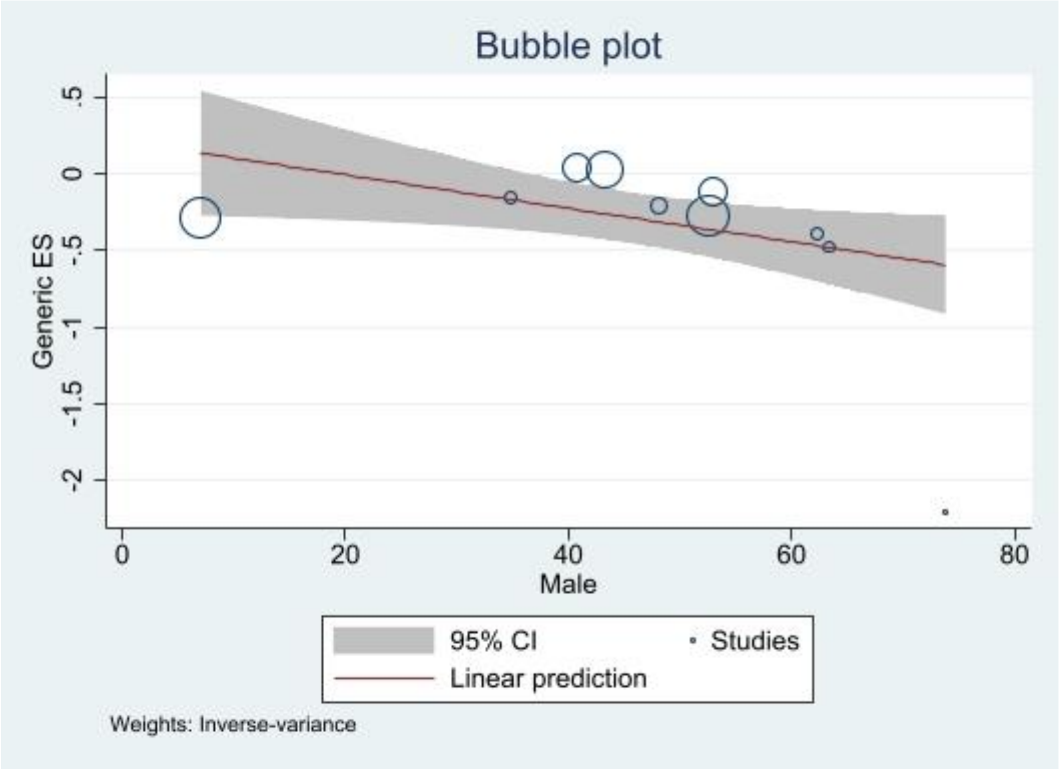

Figure S8: Meta-regression of male as an effect modifier for all-cause mortality

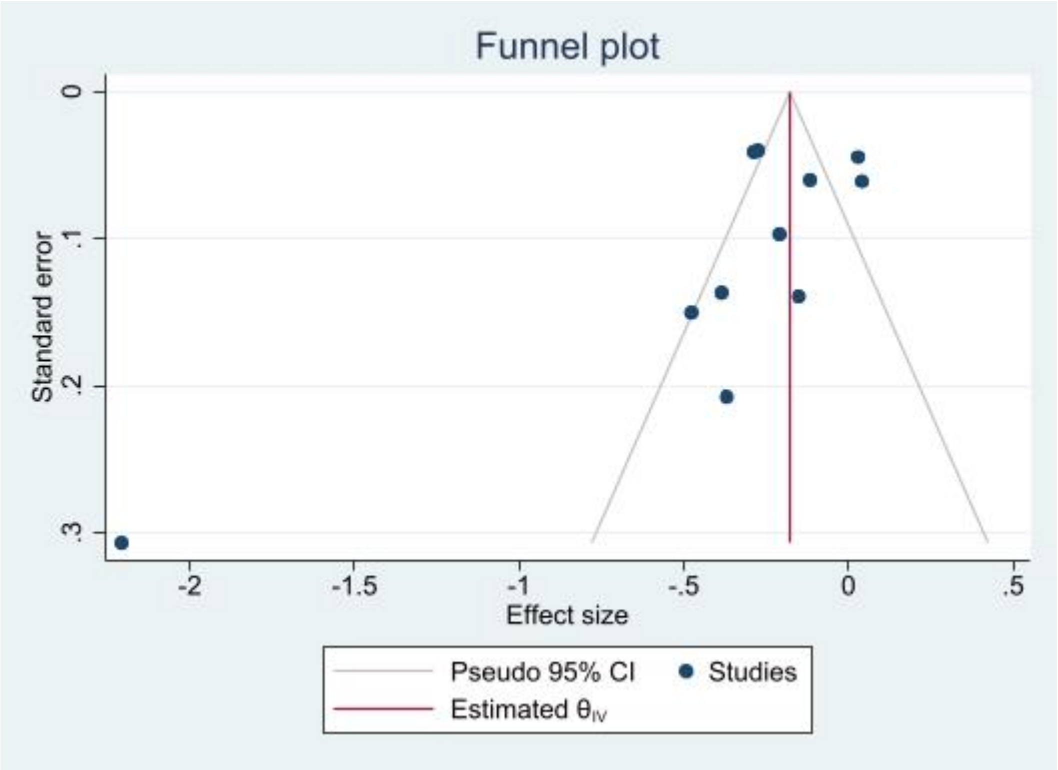

Figure S9: Funnel plots for publication bias for primary outcome including (A) all-cause mortality

A) MI

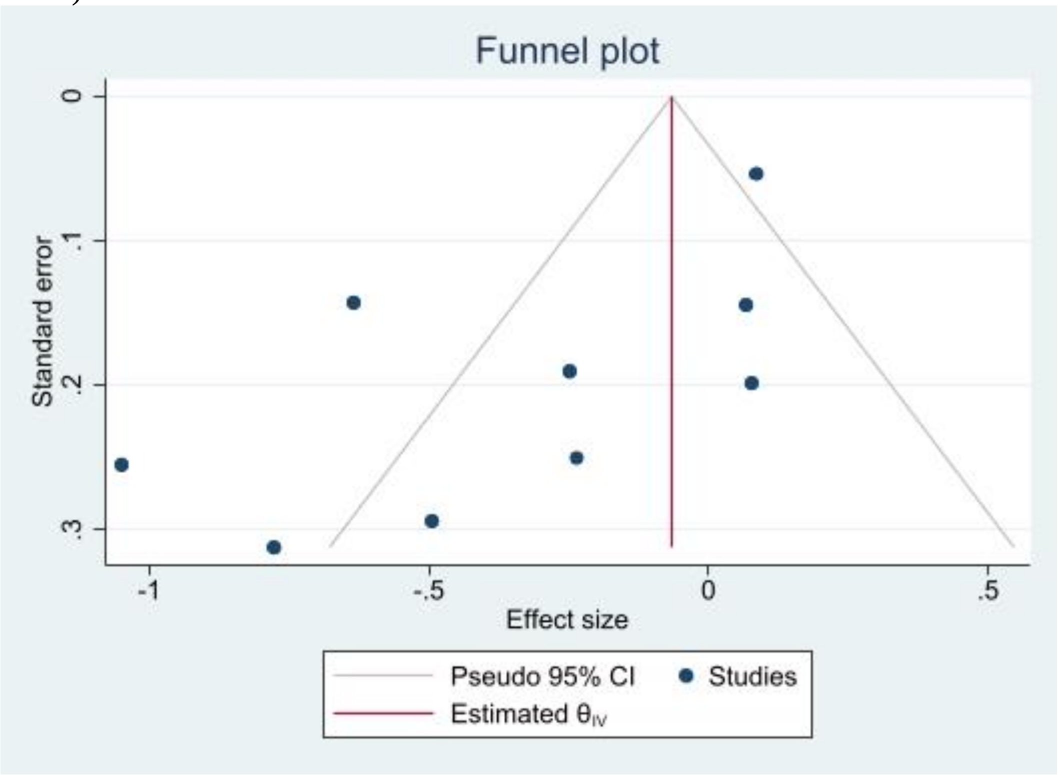

B) CV mortality

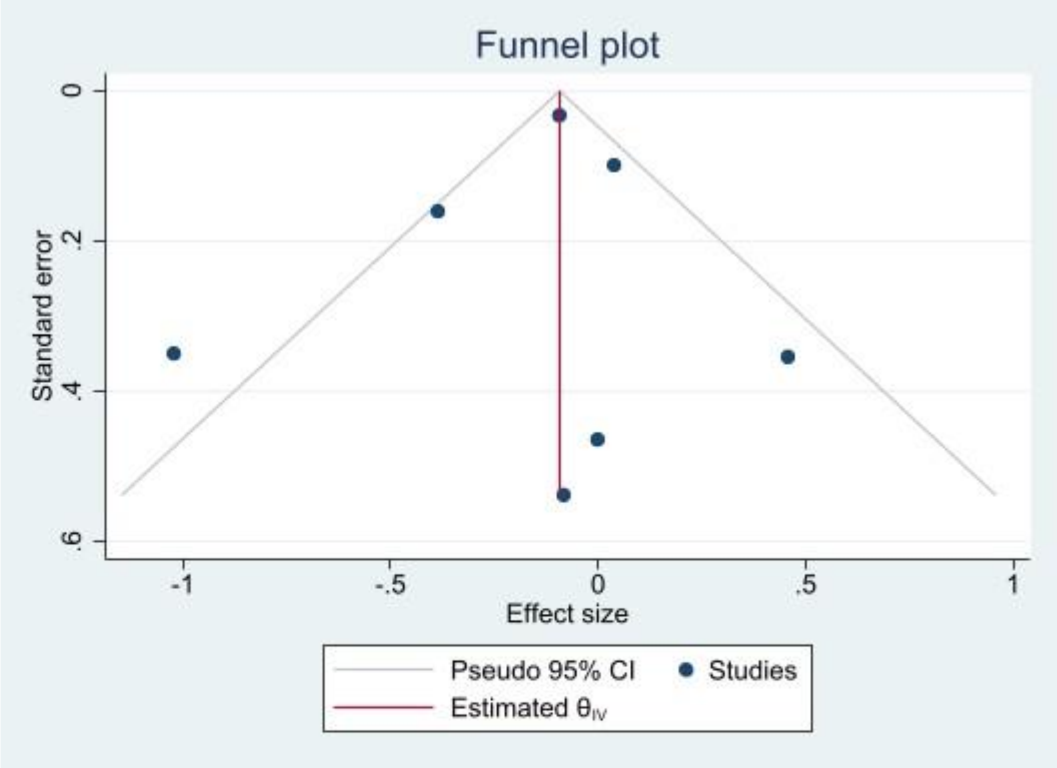

C) Stroke

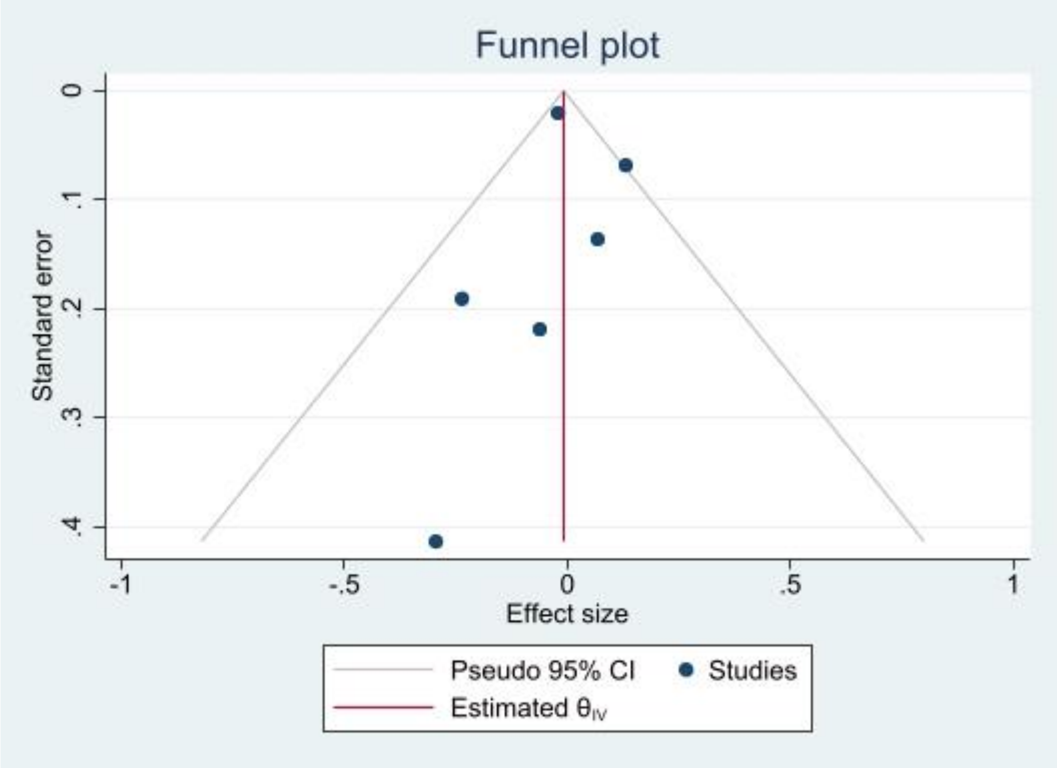

Figure S10: Funnel plots for publication bias for secondary outcomes including A) MI, B) CV mortality, C) Stroke
